# Global Mapping of Infectious Diseases: Methods, Examples, and Emerging Applications

**DOI:** 10.3201/eid1304.070037

**Published:** 2007-04

**Authors:** Martin I. Meltzer

**Affiliations:** * Centers for Disease Control and Prevention, Atlanta, Georgia, USA

In 1849, John Snow pioneered the application of mapping to public health by producing a map depicting locations of cholera cases around the Broad Street pump in London (*1*). Thus, any book describing recent advances in mapping infectious disease is potentially of interest to practicing public health officials. The topics covered in the 11 chapters in this book range from the very technical, such as descriptions of satellite-obtained environmental data, to the geographic and climatic distribution of dengue and yellow fever, plotted in risk maps for those diseases. However, most public health officials will likely find this book overly specialized, particularly the first 4 chapters. These contain detailed descriptions of the technical aspects of measuring, modeling, and analyzing climatic and geospatial data. Public health officials are likely to appreciate the chapters describing the distribution and factors potentially affecting further spread of disease. These chapters present data on the distribution of malaria, dengue, yellow fever, soil-transmitted helminths, and tickborne diseases, and information on how global transport systems and climate changes could alter the distribution of diseases. 

Some of the authors have fallen prey to the rather regrettable tendency to address “hot topics,” such as bioterrorism and the spread of pandemics, even if such topics are somewhat outside the domain of the rest of the book. The result is that in 1 chapter there are 1 or 2 pages in which the authors briefly, and mostly uncritically, review some of the most well-known literature on these topics. Readers would have been better served had the authors of that chapter focused on vectorborne diseases, for which they are justly well known. Furthermore, even in chapters focusing on practical aspects of disease distribution, many sections contain detailed descriptions of methods that most public health officials are likely to want to skip over. Placed at the back of the book are the color plates of maps (the central feature of such a book). This placement is annoying because it makes it difficult to quickly find the figures being described in a given chapter. Overall, this book is more likely to appeal to the specialist, who will find it a useful addition to a technical library, while most public health officials will likely be better served in seeking a book containing more general descriptions of mapping infectious diseases.

## References

[1] Richardson BW. Snow on cholera. New York: Commonwealth Fund; 1936.

